# Molecular Epidemiological Analysis of the Origin and Transmission Dynamics of the HIV-1 CRF01_AE Sub-Epidemic in Bulgaria

**DOI:** 10.3390/v13010116

**Published:** 2021-01-16

**Authors:** Ivailo Alexiev, Ellsworth M. Campbell, Sergey Knyazev, Yi Pan, Lyubomira Grigorova, Reneta Dimitrova, Aleksandra Partsuneva, Anna Gancheva, Asya Kostadinova, Carole Seguin-Devaux, Ivaylo Elenkov, Nina Yancheva, William M. Switzer

**Affiliations:** 1 National Reference Laboratory of HIV, National Center of Infectious and Parasitic Diseases, 1504 Sofia, Bulgaria; lyubomiragrigorova@gmail.com (L.G.); naydenova.reneta@gmail.com (R.D.); alexandra.partsuneva@gmail.com (A.P.); gancheva.anna@gmail.com (A.G.); deshova.asi@gmail.com (A.K.); 2 Division of HIV/AIDS Prevention, National Center for HIV/AIDS, Viral Hepatitis, STD, and TB Prevention, Centers for Disease Control and Prevention, Atlanta, GA 30329, USA; ykk7@cdc.gov (E.M.C.); nvr5@cdc.gov (S.K.); jnu5@cdc.gov (Y.P.); bis3@cdc.gov (W.M.S.); 3 Department of Computer Science, Georgia State University, Atlanta, GA 30303, USA; 4 Oak Ridge Institute for Science and Education, Oak Ridge, TN 37830, USA; 5 Department of Infection and Immunity, Luxembourg Institute of Health, 4354 Luxembourg, Luxembourg; carole.devaux@lih.lu; 6 Specialized Hospital for Active Treatment of Infectious & Parasitic Diseases, 1606 Sofia, Bulgaria; ivayloelenkov@yahoo.com (I.E.); nyancheva@gmail.com (N.Y.)

**Keywords:** HIV-1, molecular epidemiology, transmission clusters, transmission dynamics, circulating recombinant forms, drug resistance, prevention

## Abstract

HIV-1 subtype CRF01_AE is the second most predominant strain in Bulgaria, yet little is known about the molecular epidemiology of its origin and transmissibility. We used a phylodynamics approach to better understand this sub-epidemic by analyzing 270 HIV-1 polymerase (*pol*) sequences collected from persons diagnosed with HIV/AIDS between 1995 and 2019. Using network analyses at a 1.5% genetic distance threshold (*d*), we found a large 154-member outbreak cluster composed mostly of persons who inject drugs (PWID) that were predominantly men. At *d* = 0.5%, which was used to identify more recent transmission, the large cluster dissociated into three clusters of 18, 12, and 7 members, respectively, five dyads, and 107 singletons. Phylogenetic analysis of the Bulgarian sequences with publicly available global sequences showed that CRF01_AE likely originated from multiple Asian countries, with Vietnam as the likely source of the outbreak cluster between 1988 and 1990. Our findings indicate that CRF01_AE was introduced into Bulgaria multiple times since 1988, and infections then rapidly spread among PWID locally with bridging to other risk groups and countries. CRF01_AE continues to spread in Bulgaria as evidenced by the more recent large clusters identified at *d* = 0.5%, highlighting the importance of public health prevention efforts in the PWID communities.

## 1. Introduction

HIV is one of the fastest evolving RNA viruses [1]. The high HIV-1 genetic diversity is a consequence of a high replication rate, errors introduced during reverse transcription due to lack of proofreading function of the viral reverse transcriptase (RT) enzyme [2], and RT-mediated recombination between two or more distinct HIV-1 genomes in an infected person [3]. Currently, HIV-1 is divided into four phylogenetically distinct groups: M (major), N (new), O (outlier), and P representing independent zoonotic events from simian immunodeficiency virus (SIV) in chimpanzees and gorillas to HIV in humans [4]. HIV-1 group M is responsible for the current pandemic and comprises 10 distinct subtypes (A, B, C, D, F, G, H, J, K, and L), at least 102 different circulating recombinant forms (CRFs) and numerous unique recombinant forms (URFs) [5]. Globally, CRFs accounted for 16.7% of all HIV-1 infections between 2010 and 2015 and are most common in places where different subtypes co-circulate [5]. Of these CRFs, CRF01_AE has the largest global prevalence comprising 5.3% of all HIV-1 infections [5].

CRF01_AE was the first CRF identified in the HIV-1 epidemic, and it was first identified in the 1980s in sex workers in Thailand [6]. However, phylogenetic analyses demonstrated that the CRF01_AE strain first originated in Central Africa in the 1970s and then was transferred to Thailand in 1980, where it became widespread [7,8]. The highest prevalence of CRF01_AE (72.8%) occurs in Southeast Asia, followed by East Asia with 47.2% [5]. In Europe, a collaborative study among 26 European countries that included 9588 samples from patients diagnosed between 2008 and 2010 showed a 3.2% prevalence of CRF01_AE [9]. Further studies conducted in Bulgaria have found a relatively high prevalence of CRF01_AE, indicating that Bulgaria probably has the highest prevalence of this strain in Europe with around 20% CRF01_AE infections [10,11,12]. Moreover, the dissemination of CRF01_AE was not evenly distributed in different vulnerable groups and regions of the country but rather prevailed among PWID in the Sofia region by 35% of HIV-1 infections [13].

While our initial study importantly identified a subpopulation infected with CRF01_AE in Bulgaria, the analysis was limited to 41 infected persons with samples collected between 1999 and 2011 [11]. Since then, the number of CRF01_AE infections has increased 6.6-fold, representing a significant public health burden and necessitating further understanding of the underlying transmission dynamics of this subtype in Bulgaria. The aim of our current study was to characterize the transmission dynamics of the CRF01_AE sub-epidemic in Bulgaria by combining and analyzing available HIV-1 epidemiological and nucleotide sequence data to infer its origin and transmission events and to help inform public health prevention strategies.

## 2. Materials and Methods

### 2.1. Study Design and Patient Samples

Following national standards consistent with guidelines of European AIDS Clinical Society, the genotypic resistance testing of HIV-1 patients in Bulgaria was performed as soon as possible after diagnosis or as a clinical follow-up after virological failure. The tests were conducted randomly, regardless of sex, age, or transmission group of the patients. Fresh, whole blood samples from individuals with HIV-1 diagnosed between 1995 and 2019 were collected during the diagnostic process and clinical follow-up at the National Reference Confirmatory Laboratory of HIV (NRCL of HIV) Sofia, Bulgaria. Demographic and epidemiological information were collected using patient self-assessment interviews following national regulations. Blood samples were linked to demographic and clinical data by using anonymous numerical codes in accordance with the ethical standards of Bulgaria as previously described [11].

### 2.2. Dataset and Sequence Analyses

Blood samples were processed for plasma aliquots that were kept frozen at −80 °C until used. Viral RNA was isolated from plasma samples using the Abbott ViroSeq HIV-1 Genotyping Test and/or QIAmp Viral RNA Mini Kit. The HIV-1 protease and part of reverse transcriptase regions of the *pol* gene was sequenced using the ViroSeq HIV-1 Genotyping Test (Abbott) using the Applied Biosystems 3130xl genetic analyzer or TruGene DNA Sequencing System (Siemens Healthcare Medical Solutions Diagnostics, Germany) and an OpenGene DNA sequencing system following the manufacturer’s protocol [13].

HIV-1 CRF01_AE subtype was defined using a set of methods, including the automated subtype tool COMET v2.2 [14], the REGA HIV-1 subtyping tool version 3.0 [15], the jumping profile Hidden Markov Model (jpHMM) [16], and SimPlot [17].

Sequence alignments were performed using the MUSCLE algorithm implemented in AliView version 1.23 [18,19] and MAFFT version 7 [20,21]. Additional editing of the alignments was performed manually. The complete dataset contained 270 Bulgarian CRF01_AE HIV-1 sequences. All Bulgarian HIV-1 CRF01_AE sequences were deposited in GenBank with the following accession numbers: EF517429-EF517431, EF517441, EF517442, EF517444, EF517448, EF517454, EF517455, EF517461, EF517463, EF517469, EF517471, EF517476, EF517477, EF517479, EF517480, EF517486, JQ259065, JQ259069, JQ259071, JQ259083, JQ259084, JQ259089, JQ259094, JQ259107, JQ259115-JQ259117, JQ259121, JQ259128, JQ259148, JQ259167, JQ259171-JQ259173, JQ259177, KJ765390, KJ765392, KJ765395, KJ765398, KJ765403, KJ765404, KJ765414, KJ765416, KJ765422, KJ765423, KJ765425, KJ765429, KJ765431, KJ765433, KJ765437, KJ765439, KJ765445, KJ765447, KJ765463, KJ765474, KJ765483, KJ765486, KJ765487, KJ765488, KJ765490, KJ765493, KJ765497, KJ765498, KJ765500, KJ765505, KJ765510, KJ765514, KJ765515, KJ765538, KJ765549, KJ765552, KJ765564, KJ765572, KJ765579-KJ765581, KJ765583, KJ765588, KJ765594, KJ765598, KJ765600, KJ765602, KJ765606, KT805898, KT805901, KT805902, KT805904, and MW196446–MW196626.

The potential origin of the HIV-1 CRF01_AE clades in Bulgaria was evaluated by phylogenetic analyses. Approximate maximum likelihood (ML) phylogenies were reconstructed using all 270 Bulgarian *pol* isolates, and 115 sequences from a BLAST search at GenBank to the Bulgarian sequences (top 10 hits for each sequence with >95% sequence identity and de-duplicated) and 1343 HIV-1 *pol* sequences from the Los Alamos database from 2019 excluding any duplicates, using the GTR nucleotide substitution model in FastTree v2.1.10 [22]. We used a local instance of Nextstrain to conduct viral dating and evolutionary rate analyses. Nextstrain uses ML analysis implemented in TreeTime [23,24] and is used for monitoring emerging outbreaks such as COVID-19 in near-real time (https://nextstrain.org/). Three HIV-1 subtype J sequences were used as the outgroup for the Nextstrain ML analysis. The total dataset for phylogenetic analysis contained 1731 HIV-1 sequences.

Identification of CRF01_AE clusters and characterization of the transmission network was done using the sequence alignment and MicrobeTrace (http://github.com/cdcgov/microbetrace) [25] at Tamura–Nei genetic distance (d) cutoffs of 0.005 (0.5%), 0.015 (1.5%), and 0.035 (3.5%) nucleotide/substitutions/site. Graphically, transmission linkages are represented by lines drawn between both nodes, where each node represents a participant’s *pol* sequence. If a participant’s *pol* sequence was linked to another according to a specific threshold, both participants were labeled as clustered. Those participants whose *pol* sequence did not link to any other participant were labeled unclustered or singletons. Categorical and numeric assortativity coefficients for selected variables among the Bulgarian cases were calculated using the Python package https://github.com/Sergey-Knyazev/attribute_assortativity [26] NetworkX (https://networkx.github.io/) [27] and thresholds of d = 0.5%, 1.0%, 1.5%, and 2.0%.

### 2.3. Statistical Analysis

Epidemiological characteristics such as sex, age, country of birth, likely country of infection, region in Bulgaria, and transmission categories were considered. The frequencies as well as percentages were analyzed by infection with CRF01_AE and other HIV-1 subtypes. The association between CRF01_AE infection and the characteristics were evaluated by Fisher’s exact test, as sample sizes were small. Multivariable analysis was performed with logistic regression with the outcome variable as subtype CRF01_AE or other subtypes. Country of origin was excluded due to low numbers of infections outside of Bulgaria. Age at diagnosis was treated as continuous variable in the multivariable analysis. Odds ratios were estimated with 95% confidence intervals (CIs). All analyses were performed in SAS v9.4 (SAS Institute, Cary, NC, USA).

## 3. Results

### 3.1. Characteristics of the HIV-1 CRF01_AE Infections in Bulgaria

**Figure 1:**
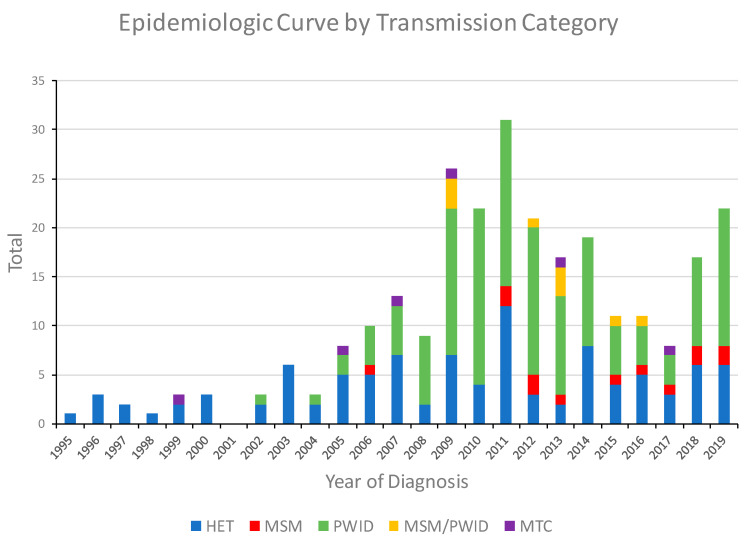
Noncumulative epidemiologic curve of HIV-1 CRF01_AE diagnoses in Bulgaria from 1995 to 2019 by transmission category. HET, heterosexual; MSM, men who have sex with men; PWID, persons who inject drugs; MSM/PWID (persons who reported both MSM and PWID); MTC, mother-to-child.

The first HIV-1 CRF01_AE infection in Bulgaria was diagnosed in 1995 in a male reporting only heterosexual behaviors, and for the next 11 years, only 33 cases with this subtype were identified, most (81.8%) of whom were heterosexual (HET). In 1999, CRF01_AE was first found in a mother-to-child transmission (MTC), and in 2002, it had spread to PWID. In 2009, 26 individuals with CRF01_AE infection were identified, 18 (69.2%) of which were PWID and 3 (11.5%) were men who have sex with men and inject drugs (MSM/PWID), leading to an outbreak among this vulnerable population in the region of the capital Sofia (Figure 1). The number of CRF01_AE infections continued to increase through 2011 with 31 cases identified that year, including 17 PWID, 12 HET, and two MSM. Following this outbreak, emergency measures were taken by the government to limit the spread of HIV in this group, and in the following years, the number of registered PWIDs and individuals from other transmission groups infected with CRF01_AE decreased. Nonetheless, starting in 2018, the number of cases began to increase again with 17 (9 PWID) in 2018 and 22 (14 PWID) in 2019.

By the end of 2019, CRF01_AE infection was found in 270 individuals, 187 (69.3%) of whom were male and 83 (30.7%) of whom were female (Table 1). The proportion of women in the group of people infected with CRF01_AE (30.7%) was statistically higher compared to individuals infected with another HIV subtype (14.4%). Age at diagnosis for persons with CRF01_AE infection ranged from 0 to 63 (mean = 30.0, median = 29.0) with the proportion of young participants (up to 19 years) twice as high (10.4%) than those infected with other subtypes (4.8%). Based on patient interviews, 251 (93%) of the infections were presumed to have occurred in Bulgaria, whereas only 19 (7%) occurred in other countries, indicating that more CRF01_AE infections occurred locally (93%) than the acquisition of HIV from another subtype (83.2%, *p* < 0.0001). With one exception of infection likely occurring in Thailand, all other cases of transmission outside the country are from Europe, including France (n = 4), Germany (n = 3), Greece (n = 2), Spain (n = 4), Serbia (n = 1), Russia (n = 1), Turkey (n = 1), and the United Kingdom (UK; n = 2). We found that participants with CRF01_AE infection had significantly lower percentages of MSM (4.8% vs. 44.6%) and higher percentages of PWID (52.2% vs. 11.2%) compared to non-CRF01_AE participants (Table 1). The percentage of HET infections with CRF01_AE was like that from other subtypes (37.4% vs. 41.9%).

Our multivariable analysis of CRF01_AE infection in Bulgaria compared to other subtypes confirmed these findings (Table 2). HIV-1-infected persons in Bulgaria were more likely to have subtype CRF01_AE compared to those residents infected in another country (OR = 1.90, 95% CI = (1.11, 3.27), *p* = 0.02). Similarly, patients in Sofia were more likely to have CRF01_AE infection compared to persons residing in other regions of Bulgaria (OR = 2.98, 95% CI = (2.19, 4.05), *p* = 0.02). Furthermore, the odds of having CRF01_AE infection in PWID compared to HET was 6.40 (95% CI = (4.41, 9.28), *p* < 0.0001) and the odds of having CRF01_AE infection in persons reporting both MSM and PWID risks compared to HET was 3.44 (95% CI = (1.64, 7.23), *p* < 0.0001). We did not observe an association of age at diagnosis with subtype infection.

### 3.2. Identification and Characterization of HIV-1 Subtype CRF01_AE Transmission Clusters in Bulgaria

**Figure 2:**
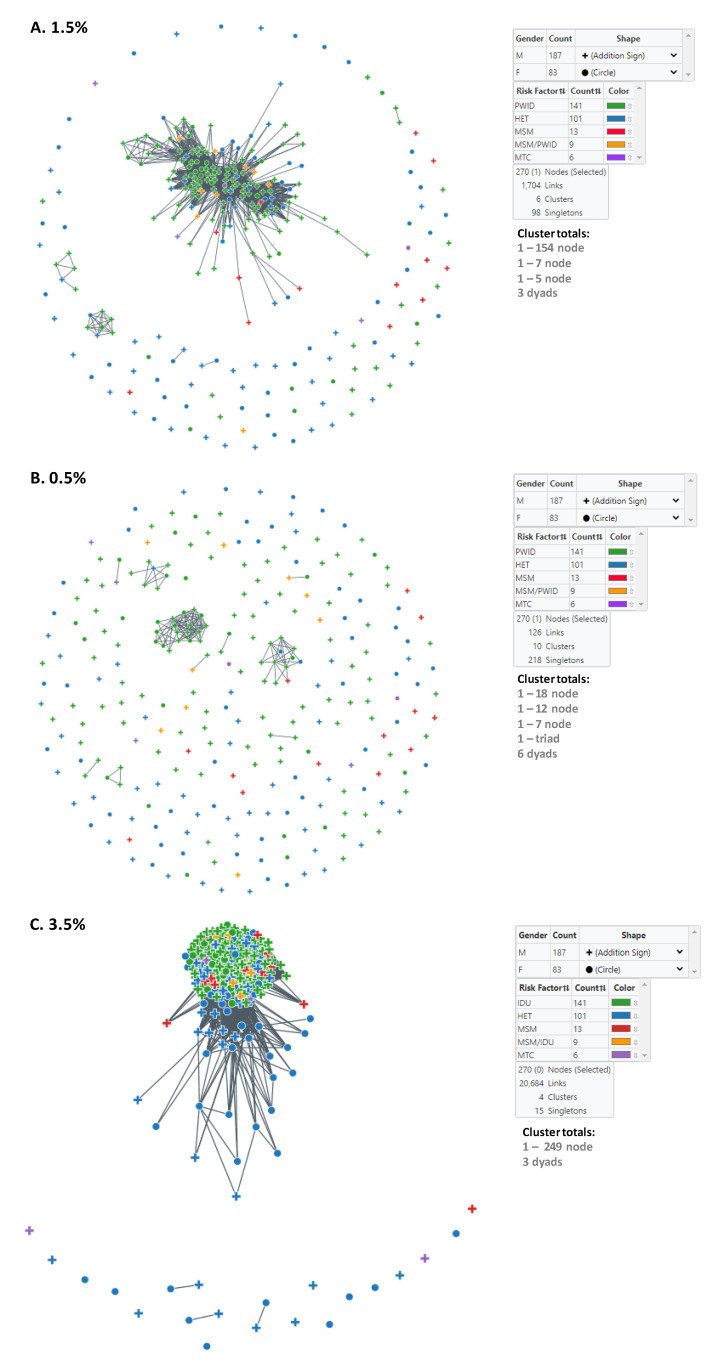
Inference of subtype CRF01-AE clusters in Bulgaria using MicrobeTrace. (**A**) Six clusters were identified using a genetic distance (*d*) of 1.5% compared to (**B**) ten clusters at *d* = 0.5%. Sex is indicated by circles (male) and addition sign (female). (**C**) Transmission network at *d* = 3.5%. Transmission category is indicated with color (red, men who have sex with men (MSM); green, persons who inject drugs (PWID); blue, heterosexual (HET); gold, persons reporting both MSM and PWID; purple, mother-to-child (MTC). Cluster totals by node (members) and total number of links in the transmission network is provided.

We used MicrobeTrace to infer HIV transmission clusters at two genetic distance thresholds to capture genetically related established and recent infections (1.5%) and then to identify more recent transmission linkage (0.5%) within Bulgaria (Figure 2). At the 1.5% cutoff, six clusters were inferred, including one large cluster containing 154 members (Table 3, Figure 2A). This large cluster consisted of mostly men (116/154, 75.3%) and PWID (108/154, 70.1%) and contained CRF01_AE-infected persons with the lowest mean and median ages at diagnosis. These results contrast with those from CRF01_AE *pol* sequences that did not cluster at this threshold, which were more evenly distributed between the sexes (57 males, 41 females), and dominated by HET (64/98, 65.3%) (Table 3). The large cluster also had slightly more individuals reporting HIV-1 infection in other countries (11/154, 7.1%) compared to (6/98, 6.1%) for *pol* sequences that did not cluster (Table 3). The large cluster also contained persons with HIV diagnoses between 2002 and 2019 (Table 3).

At the more stringent 0.5% cutoff, an additional four clusters were identified as a result of all six clusters at the 1.5% genetic distance disassociating at the lower threshold into smaller clusters or completely into singletons (Table 3, Figure 2B). The large 154-member cluster at the 1.5% genetic distance cutoff was reduced to the 18, 12, and 7 member clusters, five dyads, and 107 singletons at the 0.5% threshold. The seven-member cluster at the 1.5% cutoff with the most recent HIV diagnoses did not completely dissociate but rather was reduced to one triad, one dyad, and two singletons (Table 3). The five-member cluster and the three dyads completely dissociated. At the 0.5% cutoff, all clusters contained mostly PWID and consisted of persons diagnosed from 2009–2019 (Table 3).

Notably, cluster analysis at the 1.5% distance threshold did not capture all the Bulgarian sequences found in the large outbreak cluster identified using phylogenetic analysis described below. Once we increased the cutoff to *d* = 3.5%, most of the phylogenetic outbreak cluster sequences were in a single, large network cluster of 249 nodes (Figure 2C). All PWID were in the 249-member cluster as were most infections reportedly acquired abroad and included infections across all years of diagnosis. At this threshold, there were also three dyads and 15 singletons that were composed of mostly HETs (Table 3). The dyads were diagnosed with HIV infection between 2006 and 2019.

### 3.3. Assortative Mixing of Pairs, Sex, Similar Ages, Transmission Category, and Geographic Location in the HIV-1 CRF01_AE Transmission Networks

Assortativity is a quantitative measure often used to help characterize cluster composition that describes the likelihood that a node in a network is connected to a node bearing similar characteristics. Assortativity coefficient (r) values of 1.0 indicate perfect assortativity, while at r = −1.0, the network is completely disassortative, and when r = 0, the network is non-assortative. Our analysis found that clusters have high assortativity by region (r > 0.25), at both strict (*d* = 0.005) and relaxed (*d* = 0.015) thresholds, in most size categories and across the network in aggregate (Table 4). Region assortativity is higher for *d* = 0.005 than for *d* = 0.015 for most clusters, showing the prevalence of local transmission linkage in the network. However, region was not assortative for dyads at a strict threshold and for clusters of size 3–9 at a relaxed threshold. Sex was strongly disassortative for dyads under both threshold measures (<−0.30) and was disassortative for clusters size 3–9 at the strict threshold. Sex was not disassortative for larger clusters or the full data set. The transmission category displayed a mix of both assortativity and disassortativity, depending on the threshold and cluster size category. Dyads at strict threshold indicate a mix of transmission categories between pairs, whereas dyads at a relaxed threshold showed concurrence among transmission category between pairs. Among clusters of size 3–9, transmission category was disassortative, regardless of threshold.

### 3.4. Origin of HIV-1 CRF01_AE Infections in Bulgaria 

**Figure 3:**
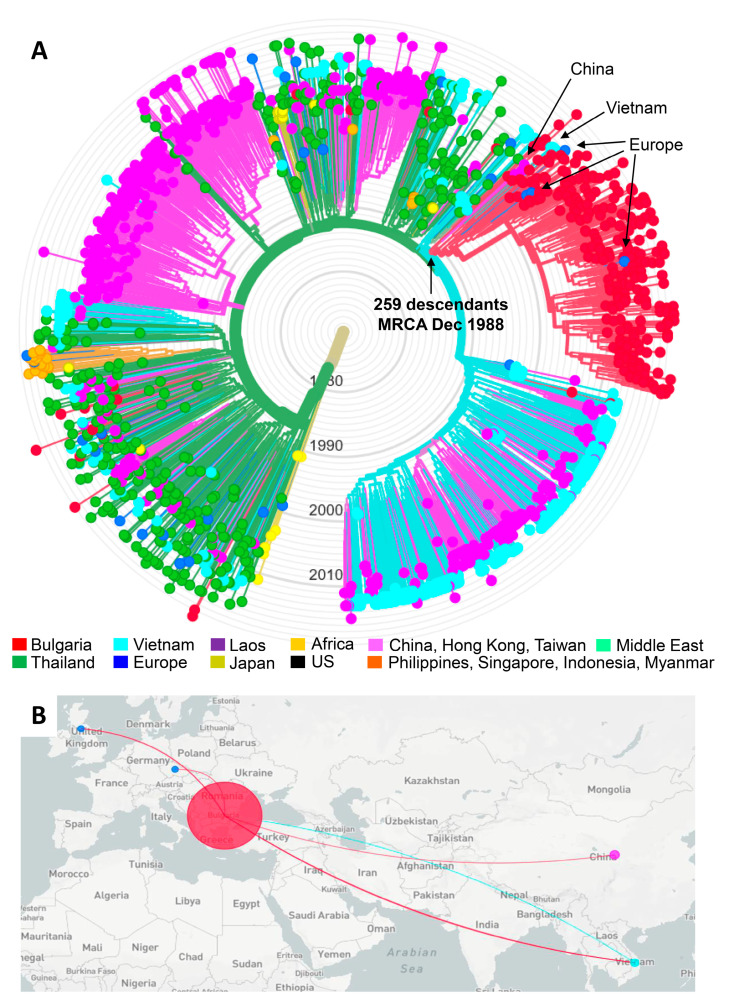
Nextstrain maximum-likelihood (ML) analysis of global HIV-1 CRF01_AE sequences. (**A**) The ML tree was constructed using 270 sequences from Bulgaria and 1458 global CRF01_AE sequences, including two from Bulgaria from a different study, 7 from Africa (Cameroon = 5, the Central African Republic = 2), 544 from China, 1 from Hong Kong, 3 from Taiwan, 20 from Europe (Belgium = 2, Czech Republic = 2, Finland = 1, France = 1, Slovenia = 1, Sweden = 7, the United Kingdom = 6), 276 from Vietnam, 49 from Laos, 362 from Thailand, 19 from the Philippines, 2 from Singapore, 3 from Indonesia, 2 from Myanmar, 6 from the United States, and two from the Middle East (Afghanistan = 1, Iran = 1). Three reference subtype J sequences were used as the outgroup. Tree branches are colored by country of birth as provided in the key. The arrows show the Bulgarian outbreak cluster showing inferred dating of the most recent common ancestor (CI, confidence interval). (**B**) Inferred geographical transmission routes with potential origins indicated by colored lines connecting countries, as provided in the (**A**).

A total of 1458 global CRF01_AE HIV-1 *pol* sequences were used to infer the phylogenetic relationships of the 270 Bulgarian CRF01_AE sequences from our study with those from other countries to evaluate potential origins of the subtype CRF01_AE sub-epidemic in Bulgaria (Figure 3). The phylogenetic tree generated by ML analysis in Nextstrain overall shows that the majority of CRF01_AE transmission events are taking place in Asia with some regional isolation after local introduction, as seen for China, but with spread and mixing in other geographic locations as for infections in Thailand and Vietnam (Figure 3A). For Bulgaria, isolated cases of CRF01_AE infection (n = 19) with HIV diagnoses between 1995 and 2019 showed phylogenetic linkage to sequences from Thailand, Vietnam, and China, and for one case, Sweden. Of these 19 cases, 16 (84.2%) reported only HET as the transmission risk factor, of which two reported acquiring infection outside Bulgaria. Two others were children infected by mother-to-child transmission, and one was an MSM.

Nextstrain ML analysis identified a large clade containing the majority of the Bulgarian CRF01_AE sequences (Figure 3A). This clade consisted of two subclades with a larger subclade clade composed of 248 Bulgarian sequences from persons diagnosed between 1996 and 2019 and 11 non-Bulgarian sequences. We refer to this larger subclade as the CRF01_AE outbreak cluster. Near the root of the outbreak cluster are four CRF01_AE sequences from China from 2005 to 2007, and three from Vietnam from 2012. Within the outbreak clade are two sequences, which were each from the Czech Republic from 2005 and the UK from 2013. The only epidemiologic data available were three of the sequences from China, which included two females and one male, all HET in their twenties [28]. The smaller subclade contained four Bulgarian sequences from persons diagnosed between 2011 and 2016 and 15 non-Bulgarian sequences.

The outbreak cluster also included two Bulgarian sequences from a man and woman with no risk behaviors identified from a previous study by a different group [29]. Most cases (64.1%, 159/248) in the outbreak clade were from the capital city of Sofia of which 108/159 (67.9%) were PWID. PWID predominated the risk factor in the outbreak clade (60.5%, 150/248), which was followed by HET (34.3%, 85/248), MSM (4.8%, 12/248), and MTC (1.6%, 4/248). 

Molecular dating inferred the most recent common ancestor (MRCA) for the outbreak clade to 12/21/88 with a confidence interval (CI) between 4/20/88 and 3/27/90. The MRCA for the small clade was estimated to have occurred on 3/11/89 (CI 6/4/88–6/10/90). The MRCA for the complete clade of 278 taxa was 8/31/88 (CI 12/2/87–11/25/89) compared to 1/18/74 (CI 9/152/71–12/22/78) for the entire CRF01_AE clade. The evolutionary rate for our CRF01_AE dataset was estimated to be 1.23E-03 nucleotide substitutions/site/year.

Figure 3B shows the inferred transmission routes for the origin and spread of the outbreak cluster using Nextstrain. The turquoise line from Vietnam to Bulgaria indicates that the original HIV-1 source of the outbreak cluster sequences in Bulgaria likely originated from Vietnam. As the outbreak in PWIDs grew in Bulgaria, HIV transmission from Bulgaria spread back to Vietnam, and to China, the UK, and the Czech Republic as indicated by the red lines from Bulgaria to these four countries. 

## 4. Discussion

In this study, we reconstructed and analyzed transmission networks of CRF01_AE, the most prevalent recombinant form of HIV-1 in Bulgaria, by combining traditional epidemiological data with HIV-1 *pol* sequences collected through 2019 and conducting phylogenetic and network analyses. Our phylodynamic approach has been used successfully before for the subtype B sub-epidemic in Bulgarian [30] and these methods are becoming standard analyses for cluster and outbreak investigations for HIV prevention [31,32,33,34]. We found that CRF01_AE infections grew into a large sub-epidemic among PWID, after it was first introduced into Bulgaria, most likely from Vietnam, with some onward transmission to other European countries. 

The first CRF01_AE infection in Bulgaria was diagnosed in 1995 mostly in HET individuals (81.8%), keeping the spread of this subtype to a limited extent. However, in 2002, CRF01_AE was introduced into PWID and then spread rapidly among this vulnerable population, leading to local outbreak in the capital Sofia in 2009 (Figure 1) [11,12,13]. Since then, CRF01_AE has become the second most predominant subtype in Bulgaria, which to our knowledge represents the highest share of CRF01_AE in Europe [5,9]. Our results resemble those described for other HIV-1 outbreaks in PWID in multiple countries, including Greece, Luxembourg, and resulting from the opioid crisis in the United States with the introduction of specific subtypes into this vulnerable population followed by rapid dissemination [16,31,35,36]. The majority (52%) of CRF01_AE infections in Bulgaria are PWID compared to only 4.8% in MSM, and PWID had at least six times greater odds of CRF01-AE infection than risk groups with other subtypes. In contrast, most persons with HIV-1 subtype B infections in Bulgaria are MSM [30]. 

We also observed that the proportion and odds of CRF01_AE infection in women was twice as high as those infected with other HIV-1 subtypes and a significantly higher percentage of these women were PWIDs (CRF01_AE 29/83 = 34.5% vs. other subtypes 14/218 = 6.4 %; *p* < 0.0001). These results correlate with the proportion of MSM being about 10 times as high in persons with non-CRF01_AE infections. Altogether, our studies show the independent introduction and spread of specific HIV-1 genotypes in different risk groups of the Bulgarian population [13,30,31]. For example, substance abuse can lead to risky sex and the further spread of HIV-1 and other sexually transmitted diseases, as we show in the current study for CRF01_AE [16,30,32,36].

Since HIV-1 has a relatively high mutation rate, different genetic distance thresholds can be used to characterize the transmission network and inform the timing of probable infection and determine whether persons are infected with genetically similar or distant viruses [37]. Network analysis was performed at different genetic distance thresholds to analyze the transmission characteristics of CRF01_AE in Bulgaria. At the 1.5% genetic distance, six clusters were identified, including one large outbreak cluster containing 154 members of which almost 41% consisted of young male PWID. These results confirm the spread of CRF01_AE infections within PWID and PWID/MSM, to a lesser extent in HET and at least MSM between 1995 and 2019. The small number of clusters (n = 3) with ≥3 sequences and the dominance of one large cluster with most participants in the study supports the development of turbulent epidemic processes which have led to an outbreak involving mostly young, male PWID. The second and third largest clusters were also predominantly PWID and men. Only 17.0% of PWIDs were singletons or dyads, and only one in nine individuals from the most vulnerable MSM/PWID population was outside clusters. In contrast, singletons (n = 98) that did not cluster at this threshold were mostly HET and more evenly distributed between the sexes, indicating likely dead-end transmission events because the person sharing the related virus died, was not identified, or for whom an HIV-1 *pol* sequence was not obtained. Another possibility is that the infection was acquired abroad, which has been reported by 6.1% of singletons with these infections reportedly acquired in the UK, Turkey, Greece, Thailand, and Spain. However, the presence of CRF01_AE among different transmission risk groups, including MSM/PWID, HET, women, and newborns inside and outside of the clusters is indicative of possible transmission of HIV-1 CRF01_AE between risk groups and likely the general population.

In order to identify and characterize more recent transmissions in the network analysis, we reduced the genetic distance threshold to 0.5%, which resulted in the formation of 10 clusters and reduced the number of participants in the large outbreak cluster to eight smaller clusters consisting of 18, 12, 7, and two (n = 5) members. Similarly, the smaller clusters found at the 1.5% genetic distance disassociated into even smaller clusters or completely into singletons at 0.5%. The clusters identified at the 0.5% cutoff contained mostly PWID and consisted of persons diagnosed from 2009 to 2019, which coincides with the period of outbreak growth in this population in Sofia (Figure 1 and Table 2). At both *d* = 0.5% and 1.5%, *pol* sequences from PWID were more likely to participate in transmission clusters, while sequences from HET and MSM were found less frequently in clusters. In general, these results suggest that although HIV-1 CRF01_AE was likely introduced into Bulgaria through HET transmission from infected persons from different countries, it then spread to PWID and continued to sustain this local sub-epidemic in Bulgaria.

Notably, we had to increase the genetic distance threshold to 3.5% to capture all taxa identified in the outbreak cluster by phylogenetic analysis. These different results likely reflect the long evolutionary history of CRF01_AE in Bulgaria over decades for which a larger genetic distance is required to detect those additional taxa in the transmission network. The 1.5% threshold used by most groups to identify relatively recent HIV-1 clusters was based on the genetic diversity of subtype B within a person over a period of about 8 years [38]. This distance threshold was later reduced to 0.5% to identify even more recent and rapidly growing clusters of concern [33,38,39]. Our cluster analysis results also suggest that non-B subtypes may require different genetic distant cutoffs to better identify transmission clusters, which is in line with other reports [40]. 

We found high assortativity by region further supporting a transmission network with more local genetic connections, which is similar to what we observed for the subtype B sub-epidemic in Bulgaria [30]. The signal of strong disassortativity by sex suggests that smaller clusters and strict thresholds seem to enrich for closely related, heterosexual pairs in Bulgaria. A mix of high assortativity (r >> 0) and high disassortativity (r << 0) was observed with respect to transmission category, especially between dyads at strict and relaxed thresholds. It is important to note that assortativity comparisons between thresholds are necessarily comparing different clusters/dyads that contain different members. For example, dyads under a strict threshold will join to form larger clusters when a relaxed threshold is applied, and this principle is repeated for clusters of any scale.

In order to reconstruct the potential origin of CRF01_AE in Bulgaria, we conducted a global phylogenetic analysis of HIV-1 CRF01_AE sequences. Our overall CRF01_AE phylogenies, MRCA divergence dates, and evolutionary rate results from the Nextstrain analysis are consistent with those in the literature, including studies using complete genomes, providing strong support for our findings [8,41,42,43]. Phylogenetic analysis showed that after leaving Africa, CRF01_AE spread to and grew rapidly in Southeast Asia, including Thailand and Vietnam and then to China [5,8,44]. From Southeast Asia CRF01_AE spread to other countries, including Bulgaria. Our analysis inferred two different Bulgarian phylogenetic groups, including a large outbreak clade of 248 Bulgarian *pol* sequences mostly from PWID. Near the root of the outbreak clade were sequences from Vietnam and China with the Nextstrain analysis inferring Vietnam as the potential origin of the other sequences in the outbreak clade. Our analyses support our previous findings using smaller numbers of both Bulgarian and Asian sequences [13] and likely reflect the international employment agreement between Bulgaria and Vietnam in 1980 and 1990 for Vietnamese workers to come to Bulgaria and which overlaps with the inferred MRCA between 1988 and 1990 for the outbreak clade. These Vietnamese immigrants lived mostly in the capital city of Sofia where the majority (60.7%) of the CRF01_AE cases were diagnosed. A total of about 35,000 Vietnamese immigrants were estimated to be in Bulgaria from 1980 to 1991, but most returned to Vietnam in 1991 after Bulgaria became an independent democracy in 1991. Combined, these results and historical information suggest a possible introduction of CRF01_AE into Bulgaria from Vietnam immigrants between 1988 and 1990 with subsequent rapid transmission via PWIDs, which is similar to other recent global outbreaks [35,36,45,46,47,48].

The outbreak clade also includes two sequences each from the Czech Republic and the UK. These findings are consistent results of a previous study on the global distribution of CRF01_AE that identified potential exports of CRF01_AE from Bulgaria to the Czech Republic [44], but we now report the dissemination of CRF01_AE from Bulgaria to the UK, China, and back to Vietnam from this outbreak cluster. A recent study of the intercontinental spread of CRF01_AE also found exportation to the UK via heterosexual transmission but from Thailand and Vietnam [44,49]. These findings could also be due to infections acquired while visiting Bulgaria, since frequent tourism occurs in Bulgaria, which is known for its beaches and mountains. Molecular dating inferred that the MRCA for the outbreak clade was late 1988, which precedes the first diagnosis of someone with this HIV-1 strain in Bulgaria by about 7 years, suggesting infections that may have been missed.

The smaller 19-person clade was located separately on the phylogenetic tree, and the MRCA for the clade was estimated to have on occurred 3/11/89 (CI 6/4/88–6/10/90), indicating early introduction into Bulgaria. These results likely reflect the risk practices of the participants at the beginning of the CRF01_AE sub-epidemic in Bulgaria from 1995 to 2007 in which the majority (75%) of the first 56 cases were HET, followed by PWID (23.2%), MTC (5.4%), and MSM (1.8%) [13]. These findings also suggest the possibility of multiple introductions of CRF01_AE into Bulgaria from Asia.

The CRF01_AE outbreak in PWIDS resulted in major public health responses by the Bulgarian Ministry of Health and NGOs, including HIV prevention and education campaigns, including a range of harm reduction services such as syringe exchange programs, condom distribution, HIV and Hepatitis testing, and other outreach activities. Opioid substitution therapy (OST) with methadone has been in place in Bulgaria since 1995. While these measures have significantly reduced HIV diagnoses in PWID, overall, the recent trend of increased infections in PWID may be explained by multiple factors. For example, funding from the Global Fund to Fight AIDS, Tuberculosis, and Malaria to combating HIV and TB in Bulgaria expired in 2015 reducing support for prevention services. The availability of OST providers in Bulgaria for PWID is limited, making it difficult to sustain. Additional public health strategies that address these limitations are required to prevent the spread of HIV-1 in PWIDs in Bulgaria. However, despite significant efforts by public health programs and NGOs to provide educational and prevention campaigns, HIV continues to be a significant burden for PWID in Bulgaria and other European countries [29,50].

Our study also has some potential limitations. Although this is the most comprehensive study of all HIV-1 subtype CRF01_AE sequences from Bulgaria through 2019, our study population included only persons for whom the HIV-1 *pol* gene was successfully obtained. As with all molecular epidemiologic studies, the inability to sequence HIV-1 from all infected persons, including from persons on treatment and with low viral loads or from persons not yet diagnosed, may affect our analyses and conclusions. Our results are also limited to the *pol* region of HIV-1. Using complete genomes may provide more accurate inference of transmission histories, especially in regions where multiple subtypes persist as in Bulgaria [43]. During the early years of the epidemic, resistance tests were not performed on all patients, some of whom may have gone abroad or died without a sample remaining for further testing, which may limit conclusions on transmission histories during this period. Some individuals in our study reported acquiring infection abroad, which may be influenced by recall bias for the potential place of infection. Samples from PWID were collected following a centralized system for registration and diagnosis of individuals with HIV in Bulgaria. Nonetheless, we cannot exclude that our results are fully representative of all HIV-infected PWID in Bulgaria.

## 5. Conclusions

We applied a detailed phylodynamics approach to better understand the molecular epidemiology of HIV-1 subtype CRF01_AE infections in Bulgaria. Our findings indicate that CRF01_AE was likely introduced into Bulgaria from Asian countries multiple times since 1995 with infections rapidly spread among PWID and their contacts. These risk behaviors continue to spread CRF01_AE subtype infection in Bulgaria. Additional funding for prevention strategies in PWID are needed and should continue to include increased testing and treatment, and pre-exposure prophylaxis.

## Figures and Tables

**Table 1:** Epidemiological characteristics of individuals infected with HIV-1 CRF01_AE compared to infection with other HIV-1 subtypes and circulating recombinant forms (CRFs) in Bulgaria.

Characteristic	Subtype CRF01_AE n (%)	Other Subtypes n (%)	*p* Value
**Total**	270	1413	
**Sex**			<0.0001
Men	187 (69.3)	1195 (84.6)	
Women	83 (30.7)	218 (15.4)	
**Age (years)**			0.001
≤19	28 (10.4)	68 (4.8)	
20–29	108 (40.0)	538 (38.1)	
30–39	90 (33.3)	493 (34.9)	
40–49	35 (13.0)	204 (14.4)	
≥50	9 (3.3)	110 (7.8)	
**Country of Birth**			0.0018
Bulgaria	269 (99.6)	1361 (96.3)	
Other country	1 (0.4)	52 (3.7)	
**Likely Country of Infection**			<0.0001
Bulgaria	251 (93.0)	1176 (83.2)	
Other country ^2^	19 (7.0)	237 (16.8)	
**Region in Bulgaria**			<0.0001
Sofia	164 (60.7)	625 (44.2)	
Other regions	106 (39.3)	788 (55.8)	
**Transmission category ^3^**			<0.0001
HET	101 (37.4)	592 (41.9)	
MSM	13 (4.8)	630 (44.6)	
PWID	141 (52.2)	158 (11.2)	
Other	15 (5.6)	33 (2.3)	

Percentages are provided in parentheses. ^2^ Other countries include France (n = 4), Germany (n = 3), Greece (n = 2), Spain (n = 4), Serbia (n = 1), Russia (n = 1), Thailand (n = 1), Turkey (n = 1), and the United Kingdom (n = 2). ^3^ HET, heterosexual; MSM, men who have sex with men; PWID, persons who inject drugs; Other includes MSM + PWID (persons who reported both MSM and PWID); mother-to-child (MTC), and blood transfusion (BLD). Fisher’s exact test was applied to calculate *p* values.

**Table 2:** Multivariable analysis of selected demographic characteristics comparing HIV-1 CRF01_AE infection to that with other subtypes in Bulgaria.

Characteristic	Odds Ratio	95% CI		*p* Value
Sex (*Women* vs. *Men*)	2.14	1.48	3.08	<0.0001
Age at diagnosis (in years)	1.00	0.99	1.02	0.73
Likely country of infection (*Bulgaria* vs. *Other*)	1.90	1.11	3.27	0.02
Region in Bulgaria (*Sofia* vs. *Other*)	2.98	2.19	4.05	<0.0001
Transmission category				<0.0001
*MSM* vs. *HET*	0.13	0.07	0.24	<0.0001
*PWID* vs. *HET*	6.40	4.41	9.28	<0.0001
*Other* vs. *HET*	3.44	1.64	7.23	0.001

CI, confidence interval. Country of origin was excluded from the analysis due to the sparseness of infections outside of Bulgaria. Age at diagnosis was treated as continuous variable in the multivariable analysis. HET, heterosexual; MSM, men who have sex with men; PWID, persons who inject drugs; Other includes MSM + PWID (persons who reported both MSM and PWID).

**Table 3:** Characteristics of HIV-1 subtype CRF01_AE clusters and unclustered persons in Bulgaria, 1995–2019, using genetic distance cutoffs of 1.5%, 0.5%, and 3.5% ^1^.

	**Cluster Sizes at 1.5%**	**Male**	**Female**	**HET**	**MSM**	**MSM/PWID**	**PWID**	**Vertical**	**Mean/Median Age at Diagnosis**	**Diagnosis Date Range**	**Likely Country of Infection (Bulgaria)**	**Likely Country of Infection (Other) ^2^**
	154	116	38	30	5	8	108	3	28.4/29.0	2002–2019	143	11
	7	5	2	2	0	0	5	0	38.9/39.0	2018–2019	6	1
	5	5	0	1	0	0	4	0	33.0/32.0	2010–2019	5	0
	Three dyads (6 total)	4	2	4	0	0	2	0	38.8/32.5	1999–2019	5	1
	Singletons (98 total)	57	41	64	8	1	22	3	31.2/31.0	1995–2019	92	6
Totals	270	187	83	101	13	9	141	6	30.0/29.0	1995–2019	251	19
	**Cluster Sizes at 0.5%**	**Male**	**Female**	**HET**	**MSM**	**MSM/PWID**	**PWID**	**Vertical**	**Mean/Median Age at Diagnosis**	**Diagnosis Date Range**	**Likely Country of Infection (Bulgaria)**	**Likely Country of Infection (Other) ^2^**
	18	10	8	0	0	0	18	0	22.5/20.5	2009–2018	17	1
	12	9	3	2	1	0	9	0	30.3/31.0	2009–2015	10	2
	7	6	1	1	0	0	6	0	27.9/29.0	2011–2015	7	0
	3	2	1	0	0	0	3	0	38.0/38.0	2018–2019	2	1
	Six dyads (12 total)	9	3	0	0	2	9	1	29.2/29.5	2009–2019	12	0
	Singletons (218 total)	151	67	98	12	7	96	5	30.7/30.0	1995–2019	203	15
Totals	270	187	83	101	13	9	141	6	30.0/29.0	1995–2019	251	19
	**Cluster Sizes at 3.5%**	**Male**	**Female**	**HET**	**MSM**	**MSM/PWID**	**PWID**	**Vertical**	**Mean/Median Age at Diagnosis**	**Diagnosis Date Range**	**Likely Country of Infection (Bulgaria)**	**Likely Country of Infection (Other) ^2^**
	249	176	73	83	12	9	141	4	30.0/29.0	1995–2019	234	15
	Three dyads (6 total)	3	3	6	0	0	0	0	30.3/30.0	2006–2019	4	2
	Singletons (15 total)	8	7	12	1	0	0	2	29.5/29.0	1998–2018	13	2
Totals	270	187	83	101	13	9	141	6	30.0/29.0	1995–2019	251	19

^1^ Clusters of sizes < 3 and singletons are grouped. MSM, men who have sex with men; HET, heterosexual transmission; PWID, persons who inject drugs; Vertical, mother-to-child transmission. ^2^ Other countries include France (n = 4), Germany (n = 3), Greece (n = 2), Spain (n = 4), Serbia (n = 1), Russia (n = 1), Thailand (n = 1), Turkey (n = 1), and the United Kingdom (n = 2).

**Table 4:** Assortative mixing of HIV-1 subtype CRF01_AE clusters in Bulgaria by region, sex, and transmission category ^1^.

	Cluster Size, Genetic Distance (*d*) Threshold, Total Persons																							
	Size	*d ^2^*	Total ^3^	Size	*d*	Total	Size	*d*	Total	Size	*d*	Total	Size	*d*	Total	Size	*d*	Total	Size	*d*	Total	Size	*d*	Total
**Cluster Characteristic**	**dyad**	**0.5**	**12**	**dyad**	**1.5**	**6**	**3–9**	**0.5**	**10**	**3–9**	**1.5**	**12**	**≥10**	**0.5**	**30**	**≥10**	**1.5**	**154**	**All**	**0.5**	**52**	**All**	**1.5**	**172**
Region	0.05			**1**			**0.41**			−0.07			**0.72**			**0.29**			**0.66**			**0.28**		
Sex	**−0.33**			**−0.5**			**−0.24**			−0.07			−0.03			0.01			−0.05			0		
Transmission category	**−0.24**			**1**			**−0.3**			**−0.2**			−0.04			0.03			−0.06			0.03		

^1^ Assortativity coefficient (*r*) values of 1.0 indicate perfect assortativity, while at r = −1.0, the network is completely disassortative, and at r = 0, the network is non-assortative. Values in bold are considered significant. ^2^ *d*, genetic distance percentage. ^3^ Total number of nodes or sequences.

## Data Availability

The sequences analyzed for this study under accession names described in the text are openly available at GenBank (https://www.ncbi.nlm.nih.gov/genbank/).
